# Lightweight aerial image object detection algorithm based on improved YOLOv5s

**DOI:** 10.1038/s41598-023-34892-4

**Published:** 2023-05-15

**Authors:** Lixia Deng, Lingyun Bi, Hongquan Li, Haonan Chen, Xuehu Duan, Haitong Lou, Hongyu Zhang, Jingxue Bi, Haiying Liu

**Affiliations:** 1 School of Information and Automation Engineering, Qilu University of Technology (Shandong Academy of Sciences), Jinan, 250353 Shandong Province China; 2 School of Surveying and Geo-Informatics, Shandong Jianzhu University, Jinan, 250101 Shandong Province China

**Keywords:** Computer science, Software

## Abstract

YOLOv5 is one of the most popular object detection algorithms, which is divided into multiple series according to the control of network depth and width. To realize the deployment of mobile devices or embedded devices, the paper proposes a lightweight aerial image object detection algorithm (LAI-YOLOv5s) based on the improvement of YOLOv5s with a relatively small amount of calculation and parameter and relatively fast reasoning speed. Firstly, to better detect small objects, the paper replaces the minimum detection head with the maximum detection head and proposes a new feature fusion method, DFM-CPFN(Deep Feature Map Cross Path Fusion Network), to enrich the semantic information of deep features. Secondly, the paper designs a new module based on VoVNet to improve the feature extraction ability of the backbone network. Finally, based on the idea of ShuffleNetV2, the paper makes the network more lightweight without affecting detection accuracy. Based on the VisDrone2019 dataset, the detection accuracy of LAI-YOLOv5s on the mAP@0.5 index is 8.3% higher than that of the original algorithm. Compared with other series of YOLOv5 and YOLOv3 algorithms, LAI-YOLOv5s has the advantages of low computational cost and high detection accuracy.

## Introduction

With the continuous application of UAVs in modern life, aerial photography technology has been widely used in various fields such as civil or military. Target detection of aerial images is one of the important parts of intelligent transportation system. Positioning and tracking of ground vehicle targets through aerial photography technology can convey and reflect the ground traffic information more clearly. And it is helpful for the construction of mature intelligent transportation system. Due to the large size of aerial images and the small and dense objects such as vehicles in the images, the detection accuracy of this detection task is low^1^. Traditional vehicle detection methods in aerial images usually adopt sliding window method, while in the process of feature extraction, fixed-size Windows and hand-crafted features often affect their detection accuracy^2^. In addition, compared with common object detection, highly complex background and variable object appearance further increase the difficulty of object detection in aerial images^3^.

Deep learning with nonlinear models has been widely used in object detection, and it can transform input data features into more abstract features. The algorithm can automatically discover the features needed for classification or detection tasks, and it has powerful representation and learning capabilities. In 2015, He et al.^4^ proposed a ResNet residual network through a cross-layer connection, which improved the network's performance, and it had no effect on error while increasing the depth of the network. In 2014, the R-CNN algorithm proposed by Girshick et al.^5^ used the proposal box extraction method to segment the input image into multiple modules and merge these modules according to the similarity information, which could obtain about 2000 candidate regions of different sizes. This is a two-stage target detection method, and it has the slower detection speed and the poorer real-time detection. Therefore, single-stage object detection method is proposed, and it can directly obtain the final output results on the original image. The YOLOv1 object detection algorithm proposed by Joseph Redmon et al.^6^ in 2015 treated the object detection task as a regression problem and removed the branch of extracting candidate boxes. Its detection speed was far faster than the two-stage object detection algorithm. The YOLOv3^7^ proposed by Joseph Redmon et al. in 2018, which used Darknet-53 with better effect as the backbone network, adopted multi-scale fusion prediction based on FPN^8^ and used three sizes of feature maps to detect objects. Bochkovskiy et al.^9^ proposed YOLOv4 in 2020, which adopted image data enhancement technology at the input end, carried out multi-channel feature fusion based on PANet^10^, and adopted CIoU as the position loss function of regression box, which had greatly improved the detection speed and detection accuracy.

For object detection in UAVs aerial images, Kim et al.^11^ proposed a channel attention pyramid method, and added a detection layer specially for detecting small targets. Transposed convolution was used to replace the upsampling, and it can effectively improve the detection accuracy of YOLO on aerial images. Wang et al.^12^ proposed a hybrid extended convolutional attention module to focus on important positions in the image and increase the receptive field, so that the improved YOLOv4 algorithm had a better effect on the detection of small targets in aerial images. In order to reduce the computation, Shen et al.^13^ proposed a lightweight backbone network based on background information module and attention mechanism module to improve the utilization of background information and significant regions in the feature extraction network. In addition, adaptive anchor-free boxes were used in the detection model to predict bounding boxes, which greatly improved the detection speed. Shamsolmoali et al.^14^ proposed an image pyramid network based on rotation equivariant convolution. The model used a single-shot detector and Lightweight Image Pyramid Module (LIPM) to extract representative features in parallel and generate regions of interest by optimization method, which improved the detection performance of small objects in aerial images. Koga et al.^15^ proposed Hard Example Mining (HEM) during the training process of a convolutional neural network, and HEM can be used for vehicle detection in aerial images. Meanwhile, HEM was applied to stochastic gradient descent (SGD) to select the most informative training data by calculating the loss value in each batch and using the example with the largest loss.

With the development of deep learning, the volume and structure of neural networks are getting larger and more complex, and the hardware resources required for training and prediction are also gradually increasing. Due to the limitations of hardware and computing power, mobile devices are difficult to train complex deep-learning network models. Since 2016, SqueezeNet^16^, MobileNet^17^, ShuffleNet^18^, NasNet^19^ and other lightweight networks have been proposed. These lightweight networks make it possible to run neural network models on hardware with lower computing power, such as mobile terminals and embedded devices.

YOLOv5, proposed by Ultralytics LLC in 2020, is also a single-stage object detection algorithm improved and upgraded based on YOLOv3. It has a more lightweight network model, faster detection speed, and better performance in detection accuracy, which is more conducive to industrial deployment and application. While, most aerial images of UAVs are smaller targets. YOLOv5 used for small target detection has lower detection performance, and is difficult to deploy on performance-constrained mobile platforms. To facilitate the deployment of the algorithm model to the hardware platform with limited performance, the paper proposes LAI-YOLOv5s used for a lightweight aerial image object detection. Its detection effect is much better than YOLOv5s and is also at the top level compared with other series of YOLOv3 and YOLOv5.

The work in this paper includes three parts:

1. In order to detect the small and many features in detection tasks, the paper proposes a new Feature Fusion Network DFM-CPFN (Deep Feature Map Cross Path Fusion Network). The medium-size detection head in the original algorithm is replaced by the enormous-size detection head after two upsampling operations, and then it is fused with the features in the backbone network respectively, which enriches the location information of the in-depth features.
2. In order to solve the problem of gradient disappearance caused by network deepening, the paper designs a VB module based on VoVNet^20^ to improve the backbone network. On the premise of retaining the residual structure, the output of multiple convolutional layers is spliced together at the end, which better ensures the transmission of features and gradients, and avoids feature redundancy.
3. Due to the high computational cost, object detection algorithm is difficult to deploy on mobile devices with limited performance. In order to solve the problem, the paper designs the C3SFN module based on ShuffleNetV2^21^, which can make the improved algorithm model more lightweight. The computational cost of the algorithm is effectively reduced.

### YOLOv5s methods

**Figure 1:**
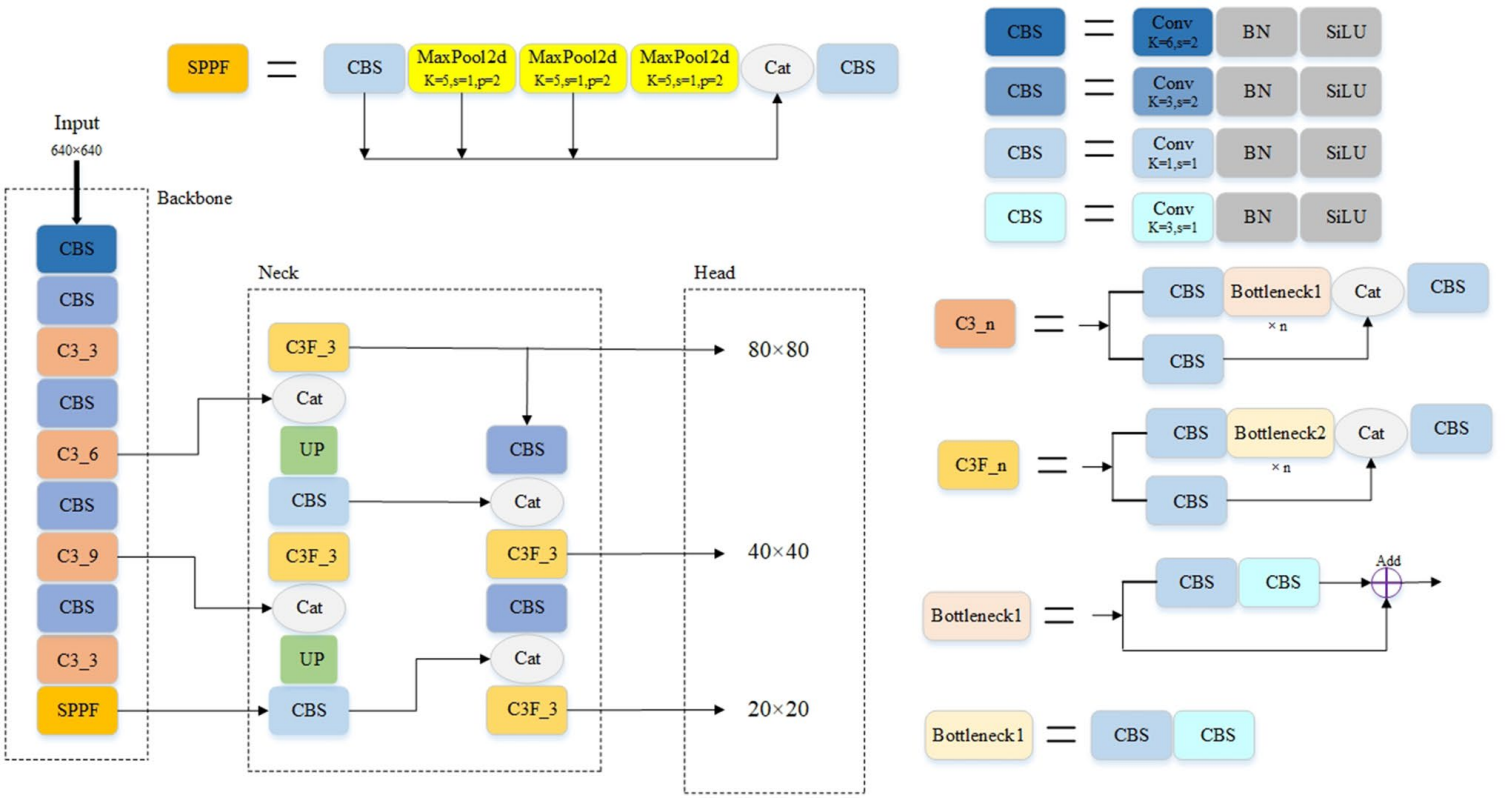
Network structure diagram of YOLOv5s.

Figure 1 shows the network structure diagram of YOLOv5s. YOLOv5 is divided into multiple series such as s, m, and l by controlling the depth and width of the network, and their differences are only in the different scaling multiples. The series with a profound or vast network has relatively good detection effect, but the computational cost is also relatively high. The series with a shallow network has significantly reduced computational cost and faster detection speed, but detection effect is relatively poor. Based on the idea of CSPNet^22^, the C3 module is added in YOLOv5s backbone network, which divides the feature map into two paths and uses the cross-stage hierarchical structure to merge. The new network architecture realizes richer gradient combinations while reducing the amount of calculation. The SPPF module with a spatial pyramid pooling structure borrowed the idea of SPPNet^23^. The neck network of YOLOv5 performs multi-scale feature fusion based on PANet. Compared with FPN, PANet adds a bottom-up feature fusion path, and its output head adds a fully connected branch to improve the quality of the prediction mask.

### LAI-YOLOv5s

**Figure 2:**
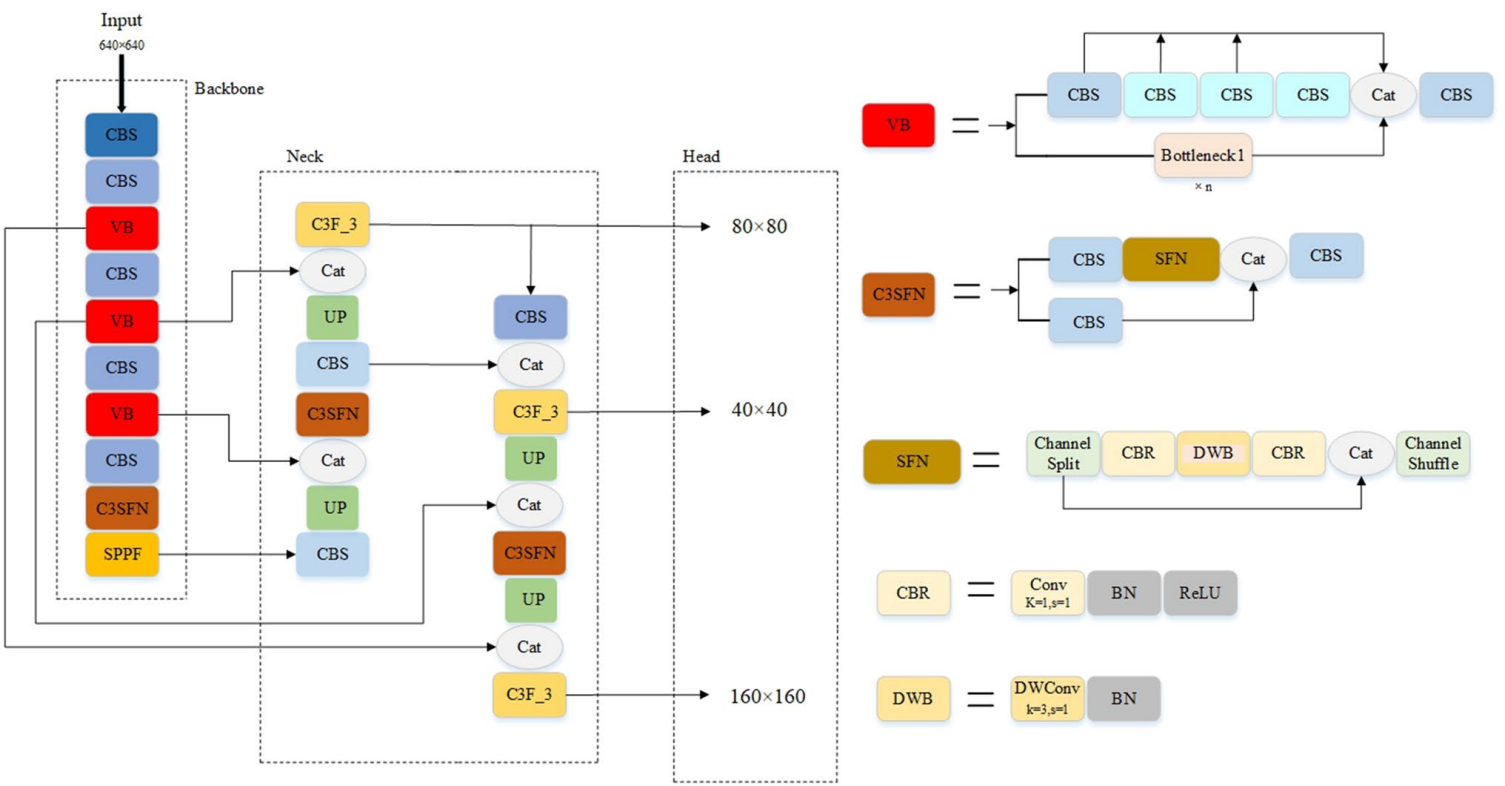
Network structure of LAI-YOLOv5s.

Firstly, a new Feature Fusion Network DFM-CPFN (Deep Feature Map Cross Path Fusion Network) is proposed, which can effectively improve the problem of profound information loss of deep features for small targets. In addition, based on VoVNet and ShuffleNetV2, the paper designs two new modules, VB and C3SFN, respectively. Two new modules can improve the feature extraction performance of the backbone network, meanwhile have a lightweight network. Compared with some other object detection algorithms and other series of YOLOv5, ablation experiments show the proposed algorithm not only has a more lightweight network model, but also has better performance in detection accuracy and detection effect. Figure 2 shows the network structure of LAI-YOLOv5s.

### The improvement of feature fusion and detection head

**Figure 3:**
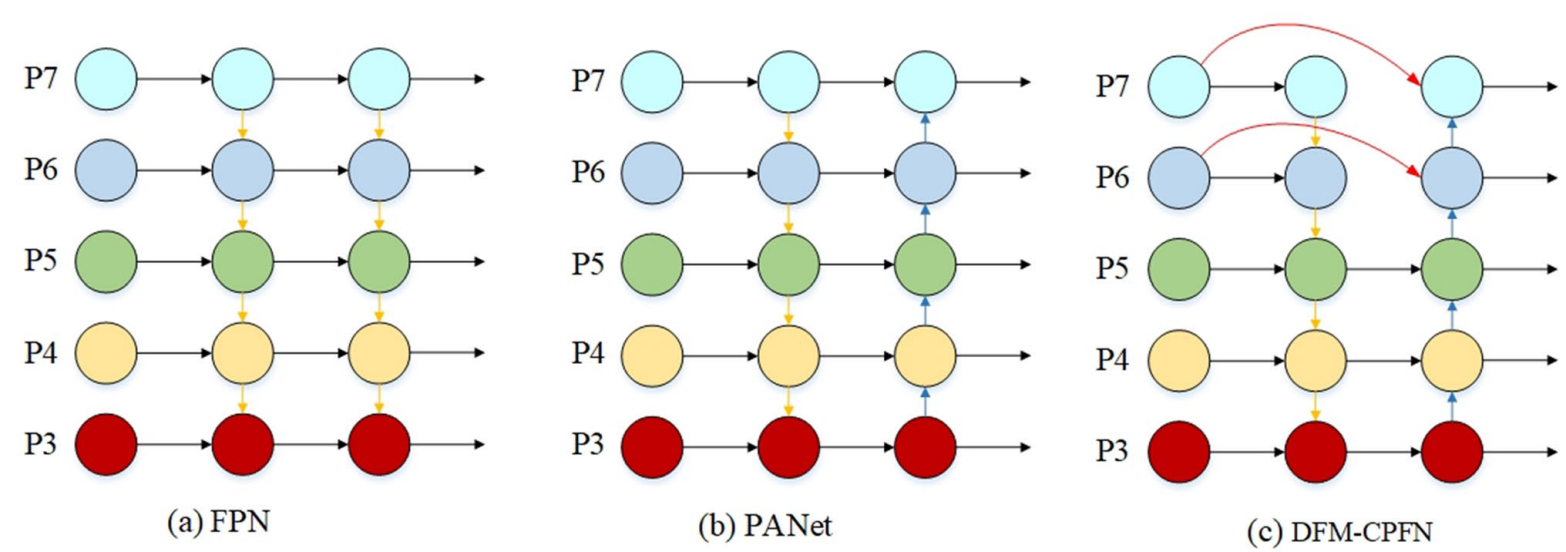
Feature fusion network structure of FPN, PANet, and DFM-CPFN.

Small and dense targets in UAV aerial images often bring great difficulties to the detection task. The PANet feature fusion method used for YOLOv5 is improved based on FPN. With the deepening of the network, semantic information is more and more abundant, but the loss of location information will be more serious. Therefore, in order to better fuse the location information of the shallow network and improve the detection effect of the algorithm, a new feature fusion path DFM-CPFN (Deep Feature Map Cross Path Fusion Network) is proposed in this paper, which fuses the most profound feature cross paths with the backbone network on the based on PANet. Figure 3 shows the feature fusion network structure of FPN, PANet, and DFM-CPFN.

**Figure 4:**
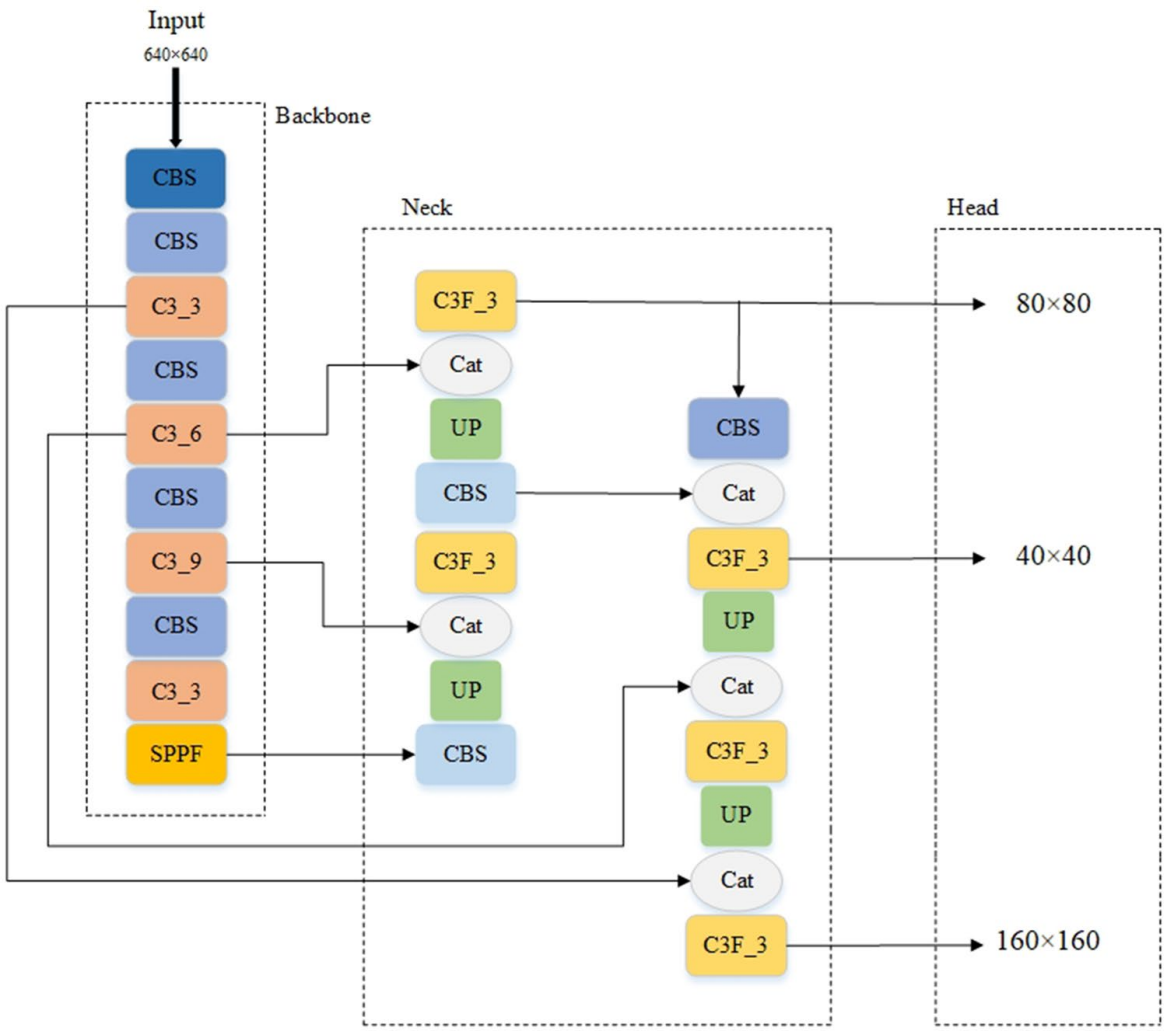
YOLOv5s network structure based on DFM-CPFN.

In this paper, the minimum size detection head in the original algorithm is replaced by the maximum size detection head, and the backbone network is fused across the path. Firstly, the path and splicing operations behind the medium-size detection head are removed, and a larger-size feature map is elicited by two upsampling operations and two C3 modules, which are used to detect smaller objects. Secondly, a cross-path fusion splice with the backbone network is added after the two up-sampling operations, which enriches the location information of the underlying features and improves the detection effect. Figure 4 shows the network structure of YOLOv5s based on DFM-CPFN.

### The improvement of backbone network

**Figure 5:**
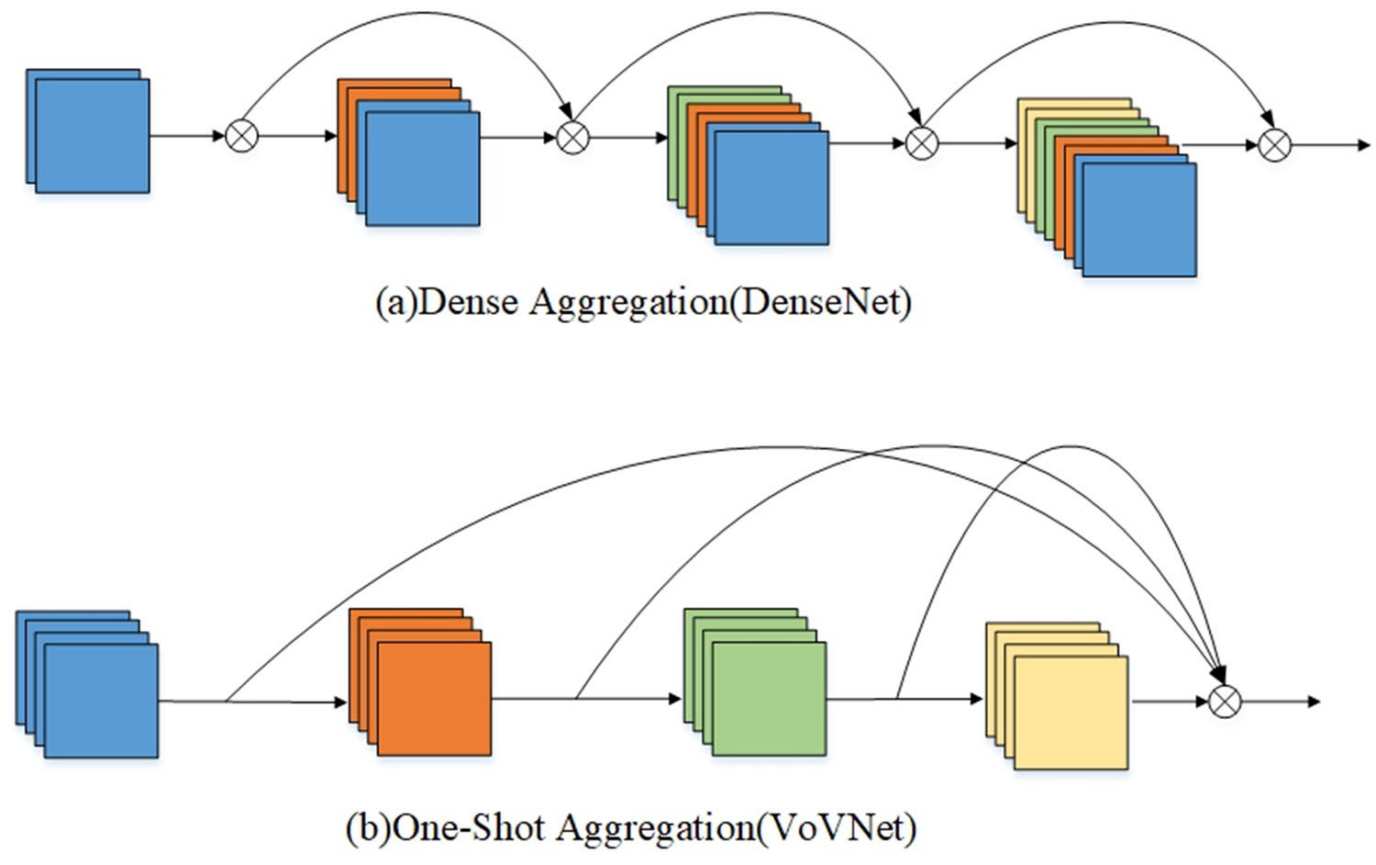
Network structure of DenseNet and VoVNet.

As the network gets more profound, the gradient vanishing problem is coursed by the gradient information passing between many layers. In 2017, Huang et al.^24^ proposed DenseNet, which input of each layer comes from the output of all previous layers. The structure makes the network narrower, and the transmission of features and gradients more effective. Therefore, the gradient vanishing phenomenon can be alleviated, but this dense connection causes the number of input channels per layer to grow linearly, which brings high memory access costs and energy consumption. Based on the OSA (One-Shot Aggregation) module proposed by Lee et al. in VoVNet, the paper proposes a VB module. VB module only aggregates all previous layers at the last layer at once, and it can effectively solve the feature redundancy caused by the dense connection of DenseNet. Figure 5 shows the network structure of DenseNet and VoVNet.

**Figure 6:**
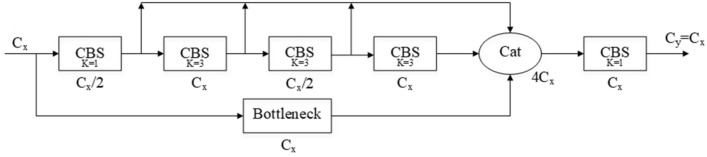
VB module network structure.

VB module designs four CBS convolution modules. The output of each convolution module is concatenated after the last module, and the Bottleneck module is retained as a separate path for concatenation. After all concatenation operations are completed, a convolution module is used to adjust the number of output channels. The VB module replaces the C3 module in the backbone network, and the network depth of 3, 6, 9, 3 is changed to 3, 3, 3, 3, which can effectively improve the degradation problem of the neural network and the gradient disappearance problem caused by network deepening. Figure 6 shows the structure of the VB module.

### The improvement of lightweight

**Figure 7:**
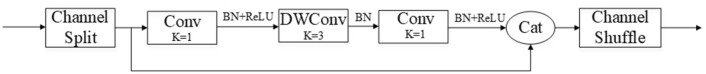
Structure of SFN module network.

FLOPs are an indirect metric that affects the computation or complexity of a network. MAC (memory access cost) can affect the inference speed of the model, and it should be considered. The paper designs an SFN module based on ShuffleNetV2, and its structure is shown in Fig. 7.

The design of SFN module mainly follows two principles. The first is to reduce the degree of fragmentation of the network. The fragmentation structure improves the model's accuracy, but it reduces efficiency. The second is to minimize element operations, such as Add, it can take considerable time, especially on GPUs. The SFN module is a bottleneck-like module. It has two branches. One branch contains two ordinary convolutions with kernel size 1 and a depthwise separable convolution with kernel size 3. The other branch is the input. And a concatenation operation fuses the two branches. Finally, a Channel Shuffle operation enables the communication between the two branches. In this paper, the Bottleneck module of the C3 module is replaced by the SFN module, and it is named the C3SFN module to replace part of the C3 modules in the original network model. Table 1 shows the comparison of the number of parameters in C3 module and C3SFN module, respectively. In the whole, the total number of parameters of C3SFN module is 55.3% less than that of C3 module in the original network. Because C3SFN adopts deep separable convolution, it can change the number of output channels of common convolution by grouping, and add a 'shuffle' operation at the end of the network. Therefore, it can effectively establish the information communication between two branches and greatly reduce the computational complexity of the original network model.

**Table 1:** Comparison of the number of parameters in C3 and C3SFN modules.

Position	Backbone				Neck				
Module	C3_3	C3_6	C3_9	C3_3	C3F_3	C3F_3	C3F_3	C3F_3	Total
Params	18,816	115,712	625,152	1,182,720	361,984	90,880	296,448	1,182,720	3,874,432

Position	Backbone				Neck				
Module	C3SFN_3	C3SFN_6	C3SFN_9	C3SFN_3	C3SFN_3	C3SFN_3	C3SFN_3	C3SFN_3	Total
Params	9,216	38,400	159,744	561,152	206,848	52,224	141,312	561,152	1,730,048
Reduction	9,600	77,312	465,408	621,568	155,136	38,656	155,136	621,568	2,144,384

## Experimental analysis

### Introduction to experimental environment

At present, UAV technology is widely used in various fields. In this paper, VisDrone2019^25^, a high-quality public dataset of UAV aerial images, is selected. AISKYEYE’s team at Tianjin University's Machine Learning and Data Mining Lab collected the dataset. The training set contains 6471 images, and the validation set has 548 images. The dataset has ten categories, and the cameras of various UAV platforms capture all images.

Hardware configuration of this experiment mainly includes Nvidia GeForce GTX3060(12 GB) graphics card, Intel Core i5 9400F processor, and 16 GB RAM. During training, the input image size is uniformly adjusted to 640 × 640.

### Experimental results of LAI-YOLOv5s

**Figure 8:**
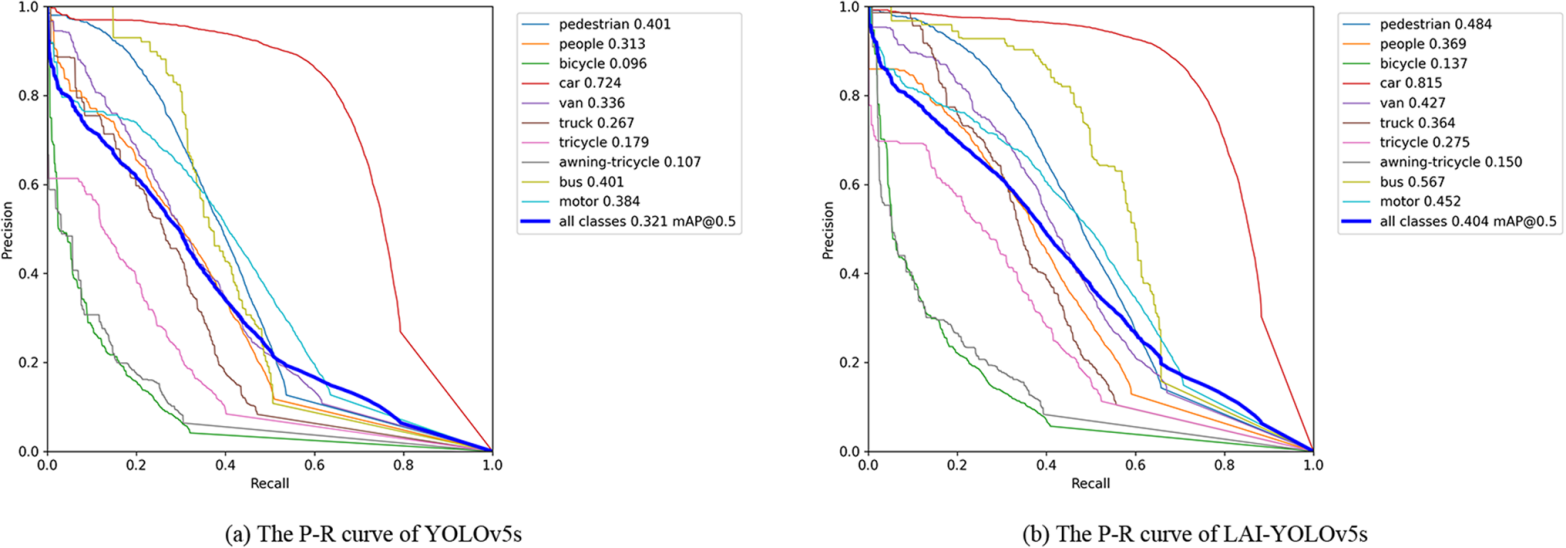
P–R curves of the two algorithms on the validation set.

Figure 8 shows the P-R curves of YOLOv5s and LAI-YOLOv5s on the validation set, where (a) is the P-R curve of YOLOv5s, and (b) is the P-R curve of LAI-YOLOv5s. Specifically, the detection accuracy of LAI-YOLOv5s with category labels of 'pedestrian', 'car', 'van', 'truck', 'tricycle', 'bus' and 'motor' has been increased by 8%. Where the performance of 'bus' is improved the most, and its accuracy is 16.6% higher than the original algorithm. In addition, the detection accuracy of LAI-YOLOv5s with category labels 'people ', 'bicycle' and 'awning-tricycle' has been improved slightly. In general, in the mAP@0.5 index of ten categories, YOLOv5s is only 32.1%, while LAI-YOLOv5s is 8.3% higher than the original algorithm, reaching 40.4%. The verification results show that LAI-YOLOv5s has a better detection effect, and its performance on mAP@0.5 is much better than the original algorithm.

**Figure 9:**
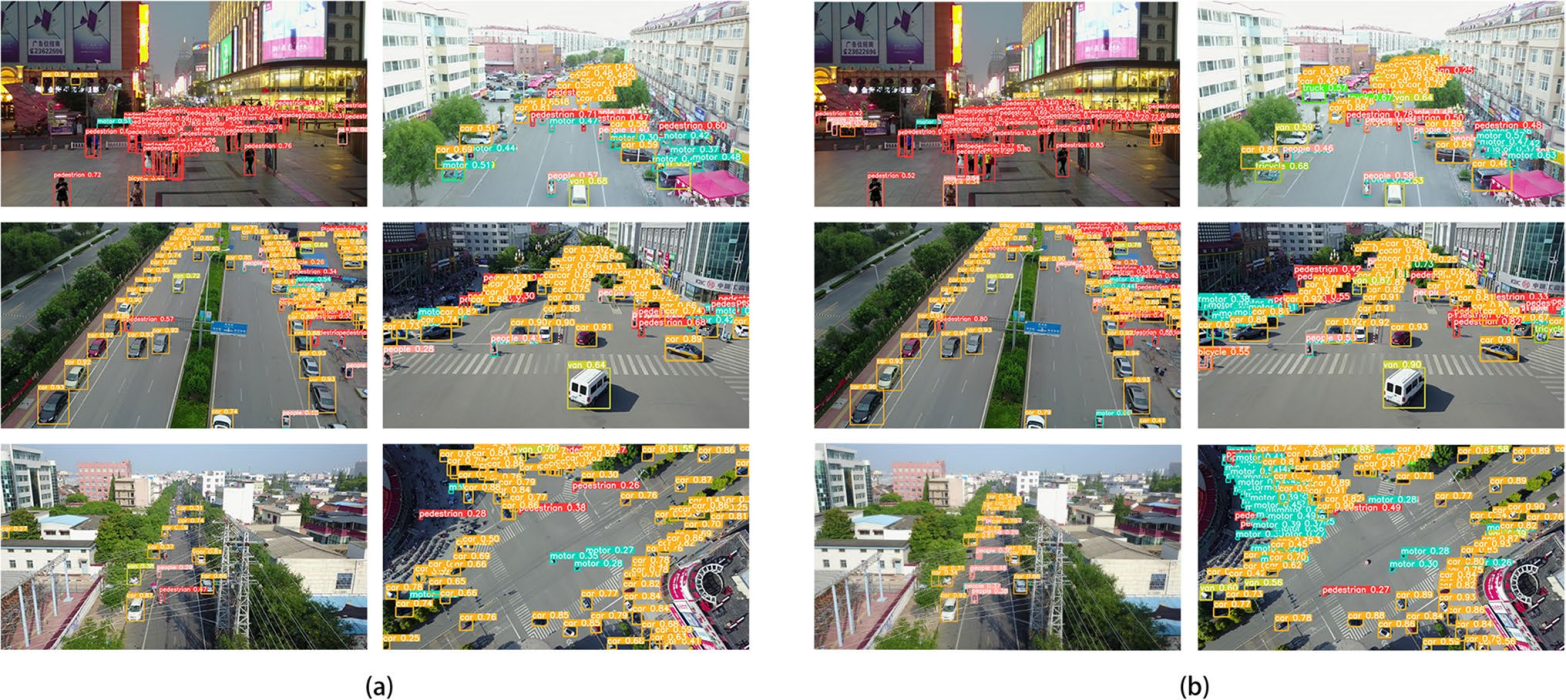
Test results of two algorithms on the test set.

Figure 9 shows the actual test results of some pictures of YOLOv5s and LAI-YOLOv5s on the test set, (a) and (b) show the test results of YOLOv5s and LAI-YOLOv5s, respectively. It can be seen that in some scenes with small or dense targets, the original algorithm is prone to the problem of false detection and missed detection. While LAI-YOLOv5s dramatically reduces the probability of false detection or missed detection, and many too-small or dense targets are detected, and their categories are identified. The excellent detection effect is attributed to the improvement of the feature fusion path and backbone network. DFM-CPFN greatly enriches the position information of deep features, and the new large-size detection head reduces the receptive field, which is more conducive to the detection of small and dense targets. In addition, the VB module effectively improves the feature extraction ability of the backbone network by concatenating all the previous layers at once in the last layer. The test results show that LAI-YOLOv5s is more effective than the original algorithm in practical applications.

### Ablation experiments

In this paper, three improved methods are proposed based on YOLOv5s. To verify the effectiveness of each method, the paper conducts a series of ablation experiments. Firstly, the improved method is compared with the original algorithm, and the mAP is used as the primary index to verify the performance of the model. Secondly, the improved algorithm is compared with other series of YOLOv5 and YOLOv3 in mAP index and lightweight index, which proves the excellent performance and practicability of the improved algorithm proposed in this paper.

**Figure 10:**
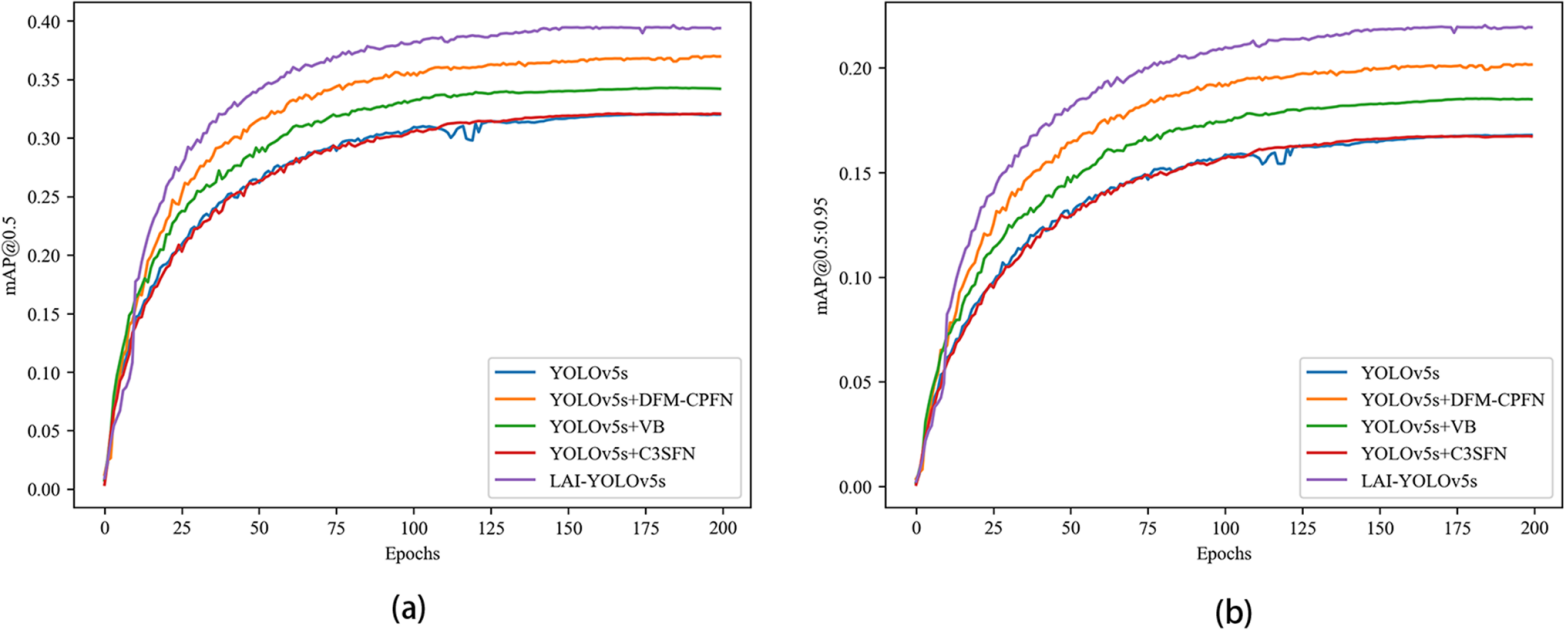
Comparison with different improved methods and YOLOv5s on mAP.

Figure 10 shows the comparison results with different improved methods and the original algorithm on the mAP index, where (a) is the comparison results on the mAP@0.5 index, and (b) is the comparison results on the mAP@0.5:0.95 indexes. It can be seen that the detection effects of two improved methods, YOLOv5s + DFM-CPFN and YOLOv5s + VB, are better than the original algorithm, and the performance of YOLOv5s + C3SFN is almost the same as the original algorithm in the mAP index. While YOLOv5s + C3SFN has lightweight network. The performance of LAI-YOLOv5s is higher than the original algorithm and all other improved methods.

**Figure 11:**
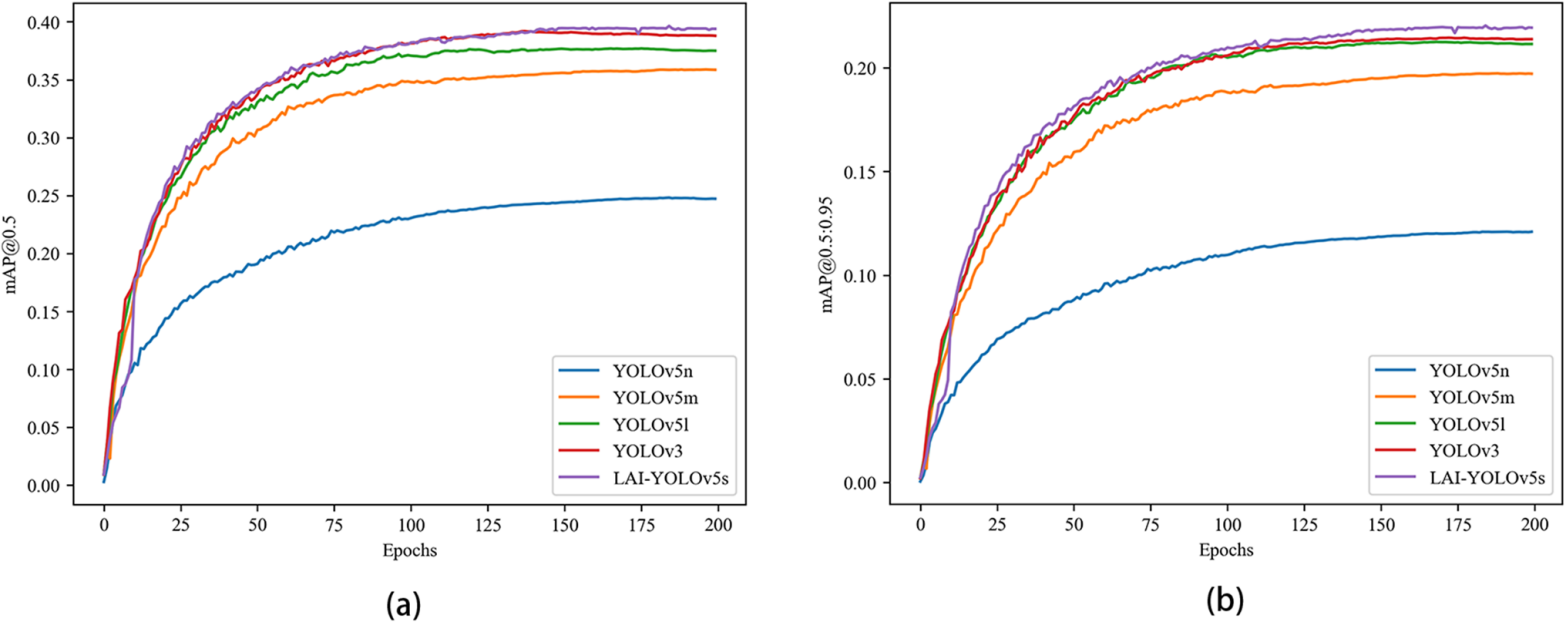
Comparison of LAI-YOLOv5s and other series of YOLOv5 and YOLOv3 on mAP.

Figure 11 compares LAI-YOLOv5s and other series of YOLOv5 and YOLOv3. It can be seen that compared with YOLOv5n, YOLOv5m, YOLOv5l, and YOLOv3 algorithms, the performance of LAI-YOLOv5s proposed in this paper is the best, and the detection effect on two mAP indicators is higher than that of other algorithms.

Table 2 shows the comparison of lightweight index between the proposed improvement and other algorithms. In this paper, ablation experiments are carried out on lightweight indicators such as parameter number, calculation amount (FLOPs), model inference time (speed), and frame rate (FPS). The method adopted by YOLOv5s + C3SFN mainly completes the light weight of the original algorithm. The number of parameters and calculation amount of YOLOv5s + C3SFN are reduced by 10% and 18.9% compared with YOLOv5s respectively, and there is a slight difference in the comparison results of model inference time and FPS. YOLOv5s + C3SFN can reduce the computational cost of the original algorithm while maintaining the accuracy of the original algorithm on mAP@0.5 is still 32.1%. YOLOv5s + DFM-CPFN and YOLOv5s + VB have negatively affected the model's lightweight, but they are within the acceptable range. These two improved methods have different degrees of improvement than the original algorithm regarding detection effect. Their mAP@0.5 indicators reached 37.0% and 34.3%, respectively.

**Table 2:** Results of ablation experiments on lightweight metrics.

Algorithm	Parameters (MB)	FLOPs (GB)	Speed (ms)	FPS	mAP@0.5 (%)
YOLOv5n	1.8	4.2	5.9	64.5	24.2
YOLOv5s	7.0	15.9	10.5	57.8	32.1
YOLOv5m	20.9	48.0	12.1	55.6	35.9
YOLOv5l	46.2	107.8	19.6	40.5	37.7
YOLOv3	61.5	154.7	22.5	36.4	39.0
YOLOv5s + DFM-CPFN	5.4	17.3	12.4	54.3	37.0
YOLOv5s + VB	14.9	34.0	12.5	52.1	34.3
YOLOv5s + C3SFN	5.2	12.9	10.6	56.8	32.1
LAI-YOLOv5s	6.3	29.0	13.2	51.3	40.4

Fusing the three improved methods is the LAI-YOLOv5s proposed in this paper. In terms of detection capability, the mAP@0.5 index of LAI-YOLOv5s is higher than that of YOLOv3 and other series of YOLOv5, reaching 40.4%, which is 8.3% higher than that of the original algorithm. In terms of lightweight, the parameter amount and calculation amount of LAI-YOLOv5s are 39.9 MB and 78.8 GB lower than those of YOLOv5l, and 55.2 MB and 125.7 GB lower than those of YOLOv3, respectively. Therefore, the computational complexity of LAI-YOLOv5s is much lower than that of YOLOv5l and YOLOv3. At the same time, LAI-YOLOv5s also performs much better than YOLOv5l and YOLOv3 in inference time and frame rate. More importantly, while LAI-YOLOv5s is very lightweight, it is also 2.7% and 1.4% higher than YOLOv5l and YOLOv3 on mAP@0.5. The detection effect is also excellent.

In addition, in order to ensure the portability and robustness of the improved algorithm, this paper provides several experiments results based on TinyPerson^26^. Table 3 shows the ablation experimental results of each algorithm on the TinyPerson dataset, and the number of parameters and calculation of each algorithm has been recorded in Table 2, so it is not shown here.

**Table 3:** Results of ablation experiments for TinyPerson.

Algorithm	Speed (ms)	FPS	mAP@0.5 (%)	mAP@0.5:0.95 (%)
YOLOv5n	5.1	135.1	15.6	4.7
YOLOv5s	7.1	113.6	17.5	5.4
YOLOv5m	10.7	78.1	19.9	6.3
YOLOv5l	14.1	63.3	20.9	6.7
YOLOv3	17.9	49.8	20.4	6.6
YOLOv5s + DFM-CPFN	6.6	100	20.1	6.2
YOLOv5s + VB	7.2	109.9	19.2	6.1
YOLOv5s + C3SFN	7.5	108.7	17.7	5.6
LAI-YOLOv5s	7.8	94.3	20.8	6.5

From the experimental results of detection speed, the inference time of LAI-YOLOv5s is 0.7 ms slower than that of YOLOv5s, and its frame rate is 19.3 slower than that of the original algorithm. However, from the experimental results of detection accuracy, LAI-YOLOv5s has a great improvement in two indicators: mAP@0.5 and mAP@0.5:0.95, which are 3.3% and 1.1% higher than the original algorithm respectively. Compared with YOLOv5l, which has the best detection effect in this experiment. The detection accuracy of LAI-YOLOv5s on two indicators is only 0.1% and 0.2% lower than that of YOLOv5l, respectively, and its detection accuracy is slightly reduced, but its calculation amount and parameter amount are 13.6% and 26.9% of YOLOv5l, respectively. The experimental results show that LAI-YOLOv5s not only has the same excellent detection accuracy as YOLOv5l, but also has extremely low computational cost, which can meet the requirements of lightweight deployment while ensuring the detection effect.

## Conclusions

In order to solve the problem of small object detection, LAI-YOLOv5s is proposed in this paper. It is improved from three aspects: feature fusion path and detection head, backbone network, and model lightweight. The improved algorithm not only effectively improves the detection accuracy of the models, but also has a lighter network structure. Experimental results based on VisDrone2019 dataset show that the detection accuracy of LAI-YOLOv5s on mAP@0.5 is not only 8.3% higher than that of YOLOv5s but also 2.7% and 1.4% higher than YOLOv5l and YOLOv3, which have better detection performance. Meanwhile, the computational cost of LAI-YOLOv5s is much lower than that of YOLOv5l and YOLOv3. Its parameters and computational complexity are 13.6% and 26.9% of YOLOv5l, 10.2%, and 18.7% of YOLOv3, respectively. LAI-YOLOv5s has a more lightweight network model that is more conducive to practical application and deployment.

At the same time, the proposed algorithm also has some limitations. The image quality acquired by UAV aerial photography is greatly affected by environmental factors. In rainy and foggy weather or night scene with insufficient light, the actual detection effect will be adversely affected. Therefore, how to improve the accuracy of object detection in complex environment will be the focus of future research.

## Data Availability

All data in this study are included in this paper.
